# Gene expression allelic imbalance in ovine brown adipose tissue impacts energy homeostasis

**DOI:** 10.1371/journal.pone.0180378

**Published:** 2017-06-30

**Authors:** Shila Ghazanfar, Tony Vuocolo, Janna L. Morrison, Lisa M. Nicholas, Isabella C. McMillen, Jean Y. H. Yang, Michael J. Buckley, Ross L. Tellam

**Affiliations:** 1 Data61, CSIRO, North Ryde, NSW, Australia; 2 School of Mathematics and Statistics, The University of Sydney, Sydney, NSW, Australia; 3 CSIRO Agriculture, Queensland Biosciences Precinct, St Lucia, QLD, Australia; 4 Early Origins of Adult Health Research Group, School of Pharmacy and Medical Sciences, Sansom Institute for Health Research, The University of South Australia, Adelaide, SA, Australia; Universitat de Lleida, SPAIN

## Abstract

Heritable trait variation within a population of organisms is largely governed by DNA variations that impact gene transcription and protein function. Identifying genetic variants that affect complex functional traits is a primary aim of population genetics studies, especially in the context of human disease and agricultural production traits. The identification of alleles directly altering mRNA expression and thereby biological function is challenging due to difficulty in isolating direct effects of *cis*-acting genetic variations from indirect *trans*-acting genetic effects. Allele specific gene expression or allelic imbalance in gene expression (AI) occurring at heterozygous loci provides an opportunity to identify genes directly impacted by *cis*-acting genetic variants as indirect *trans*-acting effects equally impact the expression of both alleles. However, the identification of genes showing AI in the context of the expression of all genes remains a challenge due to a variety of technical and statistical issues. The current study focuses on the discovery of genes showing AI using single nucleotide polymorphisms as allelic reporters. By developing a computational and statistical process that addressed multiple analytical challenges, we ranked 5,809 genes for evidence of AI using RNA-Seq data derived from brown adipose tissue samples from a cohort of late gestation fetal lambs and then identified a conservative subgroup of 1,293 genes. Thus, AI was extensive, representing approximately 25% of the tested genes. Genes associated with AI were enriched for multiple Gene Ontology (GO) terms relating to lipid metabolism, mitochondrial function and the extracellular matrix. These functions suggest that *cis*-acting genetic variations causing AI in the population are preferentially impacting genes involved in energy homeostasis and tissue remodelling. These functions may contribute to production traits likely to be under genetic selection in the population.

## Introduction

A major aim of population genetics is the identification of genetic variants and their biological effects that lead to complex trait variation within individuals. Genetic variants are also important for understanding the molecular relationship between gene function and phenotype. In agriculture, natural genetic variants are exploited for predicting and improving desirable production traits through DNA marker assisted selective breeding practices. In human health, genetic variants affecting gene function can be used to predict disease risk in human populations and this potentially can be coupled with targeted preclinical intervention strategies to minimise disease impact.

Genetic variation can directly alter the amino acid sequence of the protein encoded by a gene and thereby affect protein function or it can modify the level of mRNA expression. In the latter case the variant can lie within the coding or noncoding regions of a gene and thereby alter its transcriptional or mRNA turnover rates. Alternatively, the variant can lie outside the gene in gene promoters or distal regulatory elements such as enhancers. In particular, genetic variants contributing to complex traits within a population are often enriched in gene regulatory elements [1]. Collectively, these effects are termed *cis* effects as the causal genetic variant lies close to or within the affected gene as compared to *trans* effects caused by genetic influences arising from elsewhere in the genome that indirectly alter gene expression. The latter case is exemplified by a genetic variant altering the level of a transcription factor that then acts to regulate the expression of another gene located elsewhere in the genome. Allelic imbalance in gene expression (AI) results from a genetic variant acting in *cis* that preferentially alters the mRNA expression level of one allele. For a small group of genes, AI can also result from allele-specific epigenetic modifications. Genomic imprinting exemplifies the latter where parent of origin specific epigenetic modifications cause AI in approximately 100–300 genes in mammals [2–6]. Another cause of AI in a minor number of genes is clonally stable monoallelic expression arising from epigenetic changes [7].

Next generation sequencing technologies such as RNA-Seq simultaneously provide information on gene expression and genetic variation [8]. Single nucleotide polymorphisms (SNPs) identified in mRNAs can be used as convenient markers of the expression of each allele for individuals in a genetically diverse population. At heterozygous marker loci, AI can therefore be quantified. The causal genetic variant acting in *cis* on target gene expression is likely to be undefined but located in the vicinity of the impacted gene. Importantly, *trans*-acting effects of genetic variation equally alter the expression of both alleles of a gene. Thus, AI at a heterozygous marker locus is a definitive signature of a *cis*-acting genetic variant, genomic imprinting or clonally stable monoallelic expression.

Several analytical challenges are associated with the identification of genes with AI. (i) Most studies have been performed using species with ‘complete’ genome sequences. The draft genome assemblies for nonmodel animal species do not have complete representation of all genes, and all exons within genes, and they retain some assembly errors that collectively lead to incomplete AI information. (ii) An RNA-Seq sequence read originating from the non-reference (alternate) allele is less likely to successfully map to the correct genomic location. Consequently, selecting parameters in the read-mapping algorithm that better tolerate mismatches is therefore desirable [9]. This bias can be ameliorated by the use of longer, paired end reads. Alternatively, genomic sequence information from the same individuals can be used to correct for this intrinsic mapping bias, although this is only practical for an individual or very small populations. (iii) Following read mapping to the reference genome, SNP markers are identified at heterozygous positions. The efficiency of SNP discovery is related to the extent of genetic variation in the population and hence outbred populations are more informative. The efficiency is also dependent on the read depth of coverage. Thus, there is strong acquisition bias in the SNP discovery process for more highly expressed genes. (iv) A number of statistical issues are associated with multiple comparison corrections when there is combination of results from multiple SNPs to the level of a gene in the presence of correlations among the SNPs associated with the gene. This issue is also compounded by the variable number of informative SNPs per gene.

Previous studies have developed procedures for addressing aspects of the aforementioned challenges [9–14]. However, none of these individual approaches comprehensively addressed the multiple analytical challenges when using RNA-Seq data in the absence of genomic sequence information for the identification of loci showing AI in a population of outbred animals. In the current investigation, we used RNA-Seq data from ovine brown adipose samples from 18 individuals to identify genes showing AI. The analysis addressed multiple analytical biases and has revealed that genes showing AI are common and they are enriched for functions associated with mitochondria and lipid metabolism, as well as the extracellular matrix. These functions are particularly relevant to livestock growth related production traits.

## Materials and methods

### Animals and biological samples

All procedures involving animals were carried out with approval from the University of Adelaide Animal Ethics Committee. Pregnant ewes (n = 18) were maintained on a diet that provided 100% of their maintenance energy requirements [15] and the ewes were individually housed from 110 days post conception (dpc) in pens for two weeks before sampling. The pregnant ewes were humanely euthanased with an intravenous overdose of sodium pentovarbitone (8.2 g Lethobarb, Virbac Pty, ltd, Peakhurst, NSW, Australia) and foetuses removed and weighed at 132 ± 1 dpc (term is at 150 dpc). All efforts were made to minimize animal suffering. Samples of perirenal adipose tissue (PRAT) were collected from 18 singleton fetuses (16 female and 2 male) and then frozen in liquid nitrogen. The fetuses had a common sire and the ewes were unrelated. As RNA was prepared from tissue taken from relatively large tissue slices (~ 3 g) it is unlikely that clonal cell patches of allelic imbalance were sampled. Similarly, X-chromosome inactivation would be masked by equal contributions of both active X chromosome alleles in different cells within the tissue sample.

### RNA extraction and RNA-Seq library preparation

Total RNA was extracted from each PRAT sample (1 g), as previously described [16]. RNA quantity and integrity were assessed using the Bioanalyzer 2100 (RIN scores > 9; Agilent, Santa Clara, USA). RNA (5 μg) from each sample was used for library preparation (TruSeq RNA Sample Preparation kit v2; Illumina, San Diego, USA) and sequencing (paired end, 100 bp reads) was performed using a standard protocol for the HiSeq 2000 platform (Illumina, San Diego, USA). The 18 RNA-Seq libraries were randomly assigned to three sequencing lanes with six-fold multiplex sequencing per lane. After removal of sequencing adaptors, the reads for each sample were subjected to quality control assessments and filtering, as recommended by the manufacturer of the HiSeq 2000. The quality of the raw sequence reads was assessed using FastQC. The RNA-Seq library sizes (mean of 26,189,148 uniquely mapped paired end reads/ sample) were consistent with ENCODE recommendations [17].

### Sequence read alignment

Paired end 100 bp sequence reads were mapped to the ovine reference genome Oar v3.1 (http://www.livestockgenomics.csiro.au/sheep/) [18] using the STAR RNA-Seq aligner [19]. Parameters in the read-mapping software were adjusted to allow for mismatches representative of rates of allelic variation known to be present in the sheep population. Specifically, the maximum mismatch threshold was set to 10 and only reads aligning to unique genomic positions were used.

### SNP discovery

SNPs were identified from the mapped RNA-Seq reads using the UnifiedGenotyper tool in the Genome Analysis Toolkit [20]. Default parameters were used, with the exception of the minimum phred-scale confidence threshold (“stand_emit_conf"), which was set to 10. The best practice protocol used was “Germline SNP and indel discovery in whole genome and exome sequences”. The output variant call format file contained the genomic coordinates, reference and alternate allele nucleotides, and for each sample, read depths for both alleles and inferred genotypes at each informative locus. The reference allele was the nucleotide agreeing with the Oar v3.1 reference genome. A total of 7,631,907 potential SNPs were identified using the mapped sequence data [21].

The SNP coordinates in the ovine genome were then intersected with annotated genomic features including repeats and genes using the Galaxy intersection function [22]. SNPs were removed if present in known repeat regions, as described by the UCSC Simple Repeats track [23] and if they were not located within an ENSEMBL gene region [24]. SNPs were then retained if at least 5 of the 18 samples had 10 or more reads for both the reference and alternate alleles at heterozygous loci. This conservative approach ensured that SNPs were called using a minimum read depth, which directly reflected a minimum level of gene expression. The rigorous, progressive filtering reduced the number of informative SNPs to 24,355 (filtered SNPs). SNP filtering was undertaken using the filterVcf function within the VariantAnnotation R package (version 1.18.6). We considered how varying the minimal coverage threshold per locus impacted the number of filtered SNPs obtained, the number of genes tested for AI and the proportion of filtered SNPs that were also present in dbSNP (version 143). Minimum coverage thresholds of 5, 10, 20, 30, 50 and 100 reads for at least 5 of the 18 individuals were used. The selected minimum coverage threshold of 10 was less stringent than the value of 30 used in a recent human post-mortem population analysis of 28 tissue samples [25]. The rank orders of genes showing allelic imbalance for the different minimum coverage thresholds were highly correlated. Using this information, it was demonstrated that the minimum coverage threshold of 10 was optimum as it mitigated between loss of information due to an overly stringent filtered SNP discovery process and having sufficient reads to enable prediction of allelic imbalance in gene expression.

The unretained SNPs were not useful for a variety of reasons. (i) SNPs within reads that uniquely mapped to repeat regions in the genome may be compromised by incorrect mapping. (ii) SNPs in reads mapping outside of ENSEMBL genes due to incomplete gene annotation were not used to allow focus on well annotated genes. (iii) SNPs derived from low read depths (i.e. low gene expression abundance) were removed as they had poor statistical power to detect AI. (iv) SNPs with low representation in the population or low representation at heterozygous loci in the population were removed as they also had poor statistical power to detect AI.

### Identification of SNPs reporting allelic imbalance in gene expression

All sequence reads covering each SNP were considered and the number of reads N_A_ and N_a_ matching the reference (A) and alternate (a) alleles, respectively, were then identified. Minor reads with another alternative nucleotide at the SNP location (due to either an additional allele within the population at the same position or read sequencing and mapping errors) were ignored. For each of the 18 sheep, a genotype call of AA, aa or Aa was made at each of the 24,355 filtered SNPs. Testing for AI was restricted to samples that were heterozygous at the particular marker SNP position. Using the read count data from these latter samples, a likelihood ratio test (LRT) based on a Poisson model was used to test for AI. Specifically, it is assumed that N_A_ and N_a_ were independently Poisson distributed with means:

$$E(N_{A})=\lambda +\delta$$

$$E(N_{a})=\lambda -\delta$$

for some λ > δ ≥ 0. The null hypothesis of no AI corresponds to a zero-delta parameter, δ = 0. Defining X_A_ = Σ_*j*_ X_*j*A_ and Xa = Σ_*j*_ X_*j*a_ as the sum of read counts over sample *j* for alleles A and a respectively, the corresponding LRT statistic is:

$$X^{2}=(X_{A}+X_{a})\;\log\left(\frac{X_{A}+X_{a}}{2}\right)-X_{A}\;\log X_{A}-X_{a}\;\log X_{a}$$

and its asymptotic distribution is χ^2^ with one degree of freedom, thus allowing direct calculation of a P-value for each of the alternative SNP at a heterozygous locus. Often multiple SNP were tested in a single gene.

The P-values for multiple SNPs showing AI within an ENSEMBL gene were not independent and therefore traditional methods of correcting for multiple comparisons such as Benjamini-Hochberg false discovery rate (FDR) correction, while potentially useful, may be overly stringent [26]. This issue may be further confounded as (i) some genes contained more informative SNPs than others, (ii) longer genes may contain more SNPs than shorter genes and, (iii) some genes are more constrained in their functional tolerance for genetic variation. Consequently, SNPs were then combined at the gene level by taking the minimum of the unadjusted P-values over all SNPs within a gene. The minimum P-value for multiple SNPs within a gene may not be suitable to maintain valid interpretation of the P-values as there is also an underlying bias in gene expression. Therefore, genes were ranked for AI using down-sampling analysis (next section) to control for gene expression bias.

### Down-sampling to reduce bias in the identification of genes with AI

Genes that are more highly expressed lead to increased statistical power to detect AI as the increased read counts for each allele increase the ability to discern smaller levels of AI. As a strategy for addressing this issue, a down-sampling approach was used to induce equal statistical power for detecting AI by taking random subsamples of the data to the same read coverage level. Down-sampling at each SNP was undertaken by randomly sampling reads without replacement to a read depth D, and repeating this process k times. D was selected by comparing the estimated overall bias and overall variance for differing read depths. The latter corresponded to 5%, 10%, 15%, …, 100% quantiles of the overall expression. SNP rankings were computed after replication by calculating AI scores and hence ranks for each replicate, and combining ranks via the geometric mean over the k replicates. For most subsequent analyses, the top 1,500 ranked genes after down-sampling analysis were additionally filtered to only include genes with P< 1.5E-5 (gene based Bonferroni corrected P<0.05) after the original likelihood ratio test.

### Identification of SNPs impacting encoded protein function

The ENSEMBL Variant Effect Predictor (VEP) tool was used to identify the potential functional impacts of each identified SNP [27]. VEP provided information pertaining to amino acid changes arising from changes in DNA sequence, including synonymous and non-synonymous variants. The latter included changes at stop codon positions and frameshifts as well as the position of SNP relative to the coding sequences of transcripts.

### Functional enrichment analyses

Functional enrichment analyses were performed using two strategies. (i) Functional enrichment analyses were performed with the ranked list of genes (after down-sampling) using a Wilcoxon rank sum test implemented with the GOseq R package on 4,168 Gene Ontology (GO) terms each containing between 10 and 500 genes [28]. This enrichment test evaluated the likelihood that the ranking of genes associated with a particular ontology term was drawn randomly from the overall distribution of values. Use of the Wilcoxon rank sum test for identification of enriched GO terms was appropriate for these data as rankings of genes were reliably determined. (ii) The top ranked 1,500 genes showing AI in the down-sampled ranked list were then additionally filtered to include only genes with P<1.7E-5 (gene based Bonferroni P<0.05) from the original analysis prior to the down-sampling. This conservative process generated a list of 1,293 genes to test for functional enrichments. The background gene list for this GO enrichment analysis used the unique set of 5,810 genes identified from the filtered 24,355 SNPs that were tested for AI. Functional enrichment analysis for GO terms was then performed using GOrilla [29]. A q-value less than 0.05 (Benjamini and Hochberg method) was employed for selection of significantly enriched GO terms. The functional enrichment analyses described above were additionally performed using genes showing AI identified by using a minimum read coverage of 30 instead of 10.

The promoters of the 1,293 gene list (minimum read coverage of 10) were examined for enriched transcription factor binding sites using DAVID (q<0.05) [30]. The same gene list used in GSEA identified enriched experimental datasets from the Hallmark database (q< 0.05) [31, 32].

### Intersection of genes showing AI with imprinted genes, murine genes with monoallelic expression and ovine quantitative trait loci

Ovine imprinted genes were identified from the GeneImprint database [6]. This gene list was filtered to include only genes expressed in the brown adipose tissue samples as determined from the RNA-Seq data (≥ 5 samples each with > 10 reads/gene). The putative ovine imprinted gene list was then intersected with the ranked genes showing AI with P<1.7E-5 (i.e. Bonferroni P<0.05). In addition, the list of all putative mammalian imprinted genes from the GeneImprint database that were also expressed in the brown adipose tissue samples were intersected with ovine genes showing AI. The latter corresponded to the 1,293 genes resulting from the top ranked 1,500 genes after the downsizing analysis, which were then filtered to only include genes with a Bonferroni-corrected P<0.05 for the most significant SNP per gene in the original AI analysis.

The dbMAE database was used to intersect ovine genes showing AI with murine genes predicted to show monoallelic expression [33]. The predictions were based on a decision tree primarily involving the co-occurrence of the chromatin modifications H3K27me3 and H3K36me3 over the gene body [34]. The murine gene list was selected rather than human as the former has a more extensive list of genes due to the use of crosses of highly informative mouse genetic lines and it contains expression data for an extensive range of 23 cell lines and primary cell cultures. None of these murine samples were adipocytes or preadipocytes. Consequently, the myocyte C2C12 and C2C12_EqS cell lines were selected as brown adipocytes and skeletal muscle cells share a common progenitor cell [35, 36]. It was estimated that approximately 15% of mouse genes were monoallelically expressed [34]. The input for the intersection analysis was the 1,293 ovine genes resulting from the top ranked 1,500 genes after downsizing that were additionally filtered to only include genes with a Bonferroni-corrected P<0.05 for the most significant SNP per gene from the original analysis of AI. A hypergeometric statistical test was used to assess the significance of the list of orthologous genes overlapping both the ovine AI gene list and the murine monoallelic expression list. A P-value less than 0.05 was considered significant.

The Animal QTLdb database was used to intersect ovine genes showing AI with ovine quantitative trait loci (QTL) [37]. The input for the analysis was the 1,293 ovine genes resulting from the top ranked 1,500 genes after the downsizing analysis that were additionally filtered to only include genes with a Bonferroni-corrected P<0.05 for the most significant SNP per gene from the original analysis of AI. A total of 402 QTL for production, growth, meat and carcass traits, including traits associated with internal fat deposition were used in the analysis. The intersection analysis used the genome coordinates for the QTL and the most significant SNP for the genes showing AI, and was performed using the UCSC Table intersection function for the ovine genome [23].

### Software

As well as the software already mentioned in the Methods section, R was used for most of the analyses [38]. We also used the R package VariantAnnotation [39]. All scripts are available upon request.

## Results

### SNP discovery

**Fig 1:**
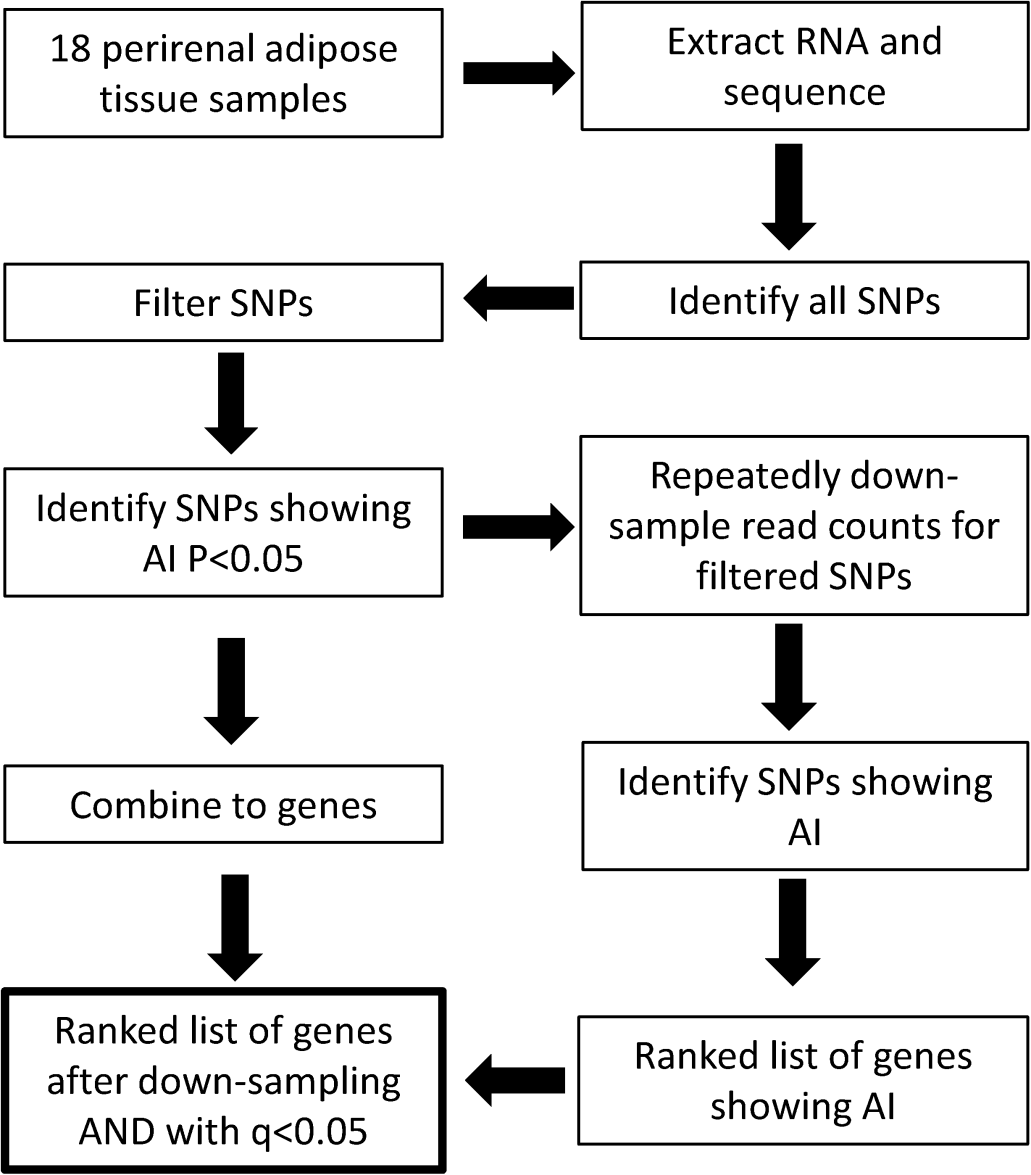
Schematic diagram of the process used for identification of marker SNPs and genes showing allelic imbalance in expression. Reads were mapped to the reference genome and potential SNPs identified. For each SNP and sample, a genotype was determined and heterozygous loci were then selected. Read counts for each genotype in these samples were then used in a Poisson model to test for AI at heterozygous loci. Genes showing AI were identified by combining multiple SNP results to the gene level via a minimum P-value. In addition, down-sampling and ranking of genes for AI was used to compensate for gene expression level bias in the discovery process. A conservative list of 1,293 genes was then identified that consisted of the top ranked 1,500 genes identified by down-sizing analysis, which was filtered to include genes with a Bonferroni P-value <0.05 for AI.

S1 Fig shows box plots for the RNA-Seq data for each of the 18 perirenal adipose tissue (PRAT) samples while S1 Table summarises the corresponding RNA-Seq library statistics. A total of ~681 million paired end reads were generated from the 18 samples. The mean library size was 37,821,376 paired end reads and a mean of 69.25% of the paired sequence reads (range 66.73–72.41%) were uniquely mapped to the ovine genome (both reads of a pair were required to uniquely map). Initially, 7,631,907 potential SNPs were identified of which 38.5% were also independently present in dbSNP [40]. Fig 1 shows a schematic representation of the process used to identify SNPs and genes showing AI at heterozygous loci. SNP filtering involved several steps including the selection of SNPs that were present in ENSEMBL genes and within these, genomic loci that were heterozygous in at least 5 of the 18 individuals. This process led to the identification of 24,355 filtered SNPs (S2 Table). To validate the filtered SNP, they were intersected with ovine SNPs independently listed in dbSNP, which were derived from different animals using different discovery technologies [40]. A large percentage (88.4%) of the filtered SNPs were independently present in dbSNP compared to 38.5% for the unfiltered SNPs. Progressively increasing the minimum read coverage threshold from 5 to 100 at heterozygous loci for at least 5 of the 18 individuals (a minimum read coverage of 10 was used above) markedly reduced the number of filtered SNPs (33,747 to 2,719) and the number of genes tested for AI (7,033 to 931) but had minor effect on the percentage of the filtered SNPs also present in dbSNP (85.4 to 92.4%) (S2 Fig). Moreover, the rank orders of genes for AI (see below) with the different minimum coverage thresholds were highly correlated e.g. R^2^ = 0.94 for gene rankings using minimum coverage thresholds of 10 and 30. Thus, the selected minimum read coverage threshold of 10 mitigated between excessive stringency in SNP discovery with consequent loss of information and the need for sufficient reads to enable reliable identification of genes showing AI. Using the minimum coverage threshold of 10 resulted in the identification of 24,355 heterozygous loci (in at least 5 of 18 samples) that were present within 5,810 genes.

Among the 24,355 marker SNPs identified at heterozygous loci, 13,881 were synonymous variants and of the remaining 10,474 SNPs, 4,831 and 3,470 were missense variants and 3' untranslated region variants, respectively (Table 1). Notably, there were 18 stop codon gains and 6 stop codon losses as well as 8 variants that affected splice sites, which collectively have potential for substantial functional impacts. The “upstream” and “downstream” variants, together representing only 2.5% of the SNPs, were due to small differences in ENSEMBL gene annotation versions used for SNP filtering (ENSEMBL Genebuild December 2013) and SNP functional impact assessments (ENSEMBL Genebuild Update May 2015), particularly the updating of a minor number of genes with previously unannotated exons.

**Table 1:** SNP categories.

SNP type^1^	SNP number
synonymous variant	13,881
missense variant	4,831
3 prime UTR variant	3,470
5 prime UTR variant	314
intron variant	70
stop gained	18
stop lost	6
stop retained variant	7
splice acceptor variant	5
splice donor variant	3
upstream gene variant	189
downstream gene variant	423
other	1,138
Total	24,355

1 The ENSEMBL Variant Effect Predictor (VEP) tool was used to identify the potential functional impacts of the identified SNPs in transcripts. UTR, untranslated region; other, SNPs with multiple annotations potentially impacting mRNA/ protein structure or function.

### Identification of heterozygous loci showing allelic imbalance in gene expression

Using the set of 24,355 filtered SNPs present in 5,810 genes, the Poisson-based likelihood ratio test (LRT) was applied resulting in 10,892 significant SNPs (unadjusted P-value <0.05) showing AI in at least 5 of the 18 samples (S3 Table). There was an average of 4.2 tested SNPs per gene over all of the data and hence there was a lack of independence of many of the tested SNPs. Consequently, SNPs were combined at the gene level by taking the minimum of the unadjusted P-values over all SNPs within a gene. Using these gene-level P-values, a total of 3,824 genes were identified at P<0.05 of which 1,580 genes were significant at P<2E-6 (gene based Bonferroni P<0.05). For the top ranked 30 genes, 25 had more than one informative SNP and of these there was an overall mean AI concordance of 80.3% for the multiple SNPs representative of each of these genes.

### Down-sampling to reduce bias in the identification of genes with AI

**Fig 2:**
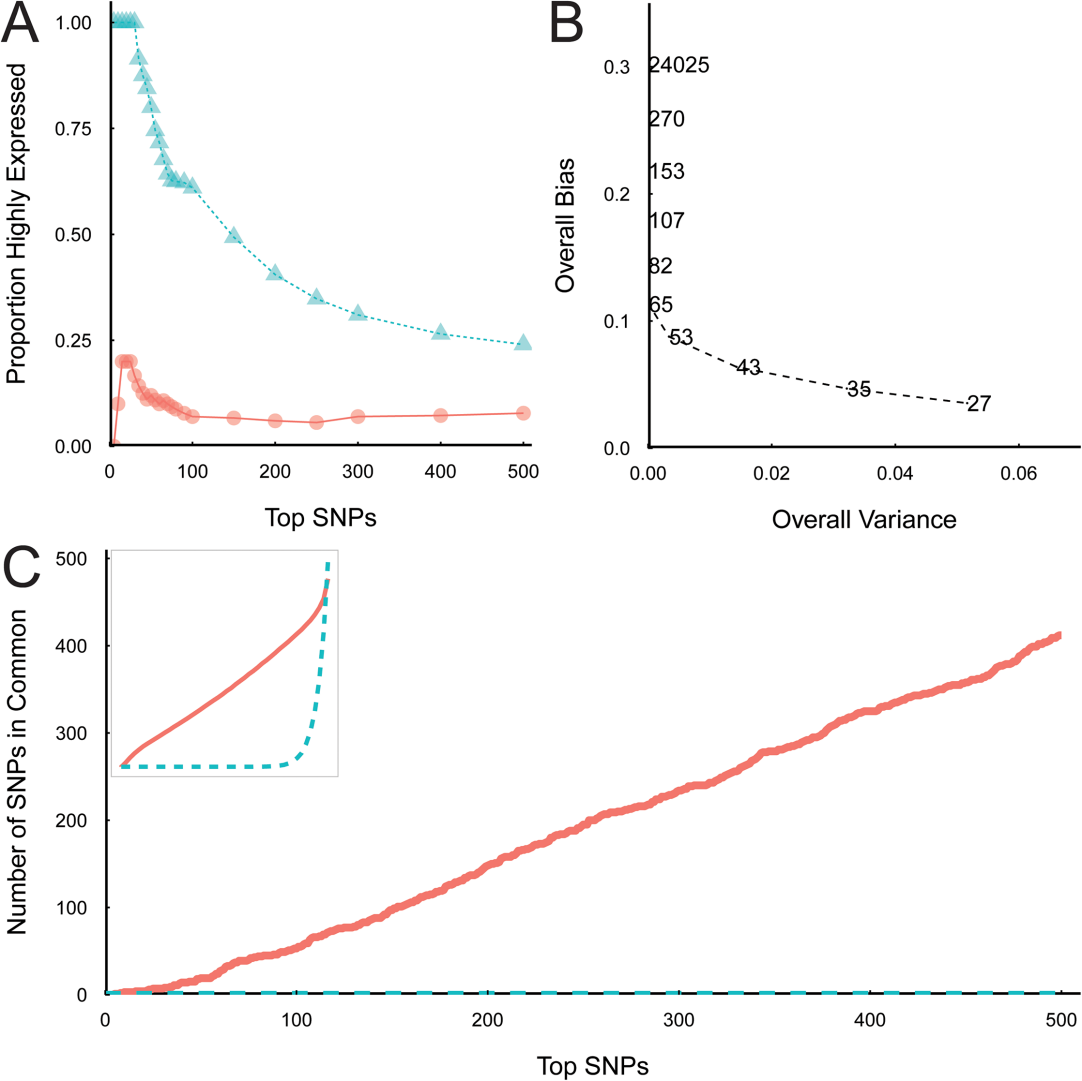
SNPs in highly expressed genes are ranked more highly for significant AI in the absence of down-sampling. A. The proportion of SNPs that are in highly expressed genes (top 5%) within the top-ranked SNPs showing AI versus size of top ranked SNP list (TopSNPs). Results without down-sampling are shown by blue triangles; results with down-sampling (D = 50, k = 20) are shown by orange circles. SNPs were considered to be highly expressed if they were greater than the 95% quantile of the overall gene expression associated with the filtered SNPs. It is expected that overall approximately 5% of the top-ranking SNPs for AI should be associated with highly expressed transcripts. B. Overall bias and variance for different values of D are shown. Overall bias was estimated by the absolute Spearman rank correlation between AI P-values (negative log-transformed) and gene expression over all samples. Overall variance was measured as the median over SNPs of the variance for k = 10 repetitions of down-sampling. Different values of D are shown on the figure panel. C. The stability of down-sampling compared to a random selection of SNPs is shown. For each number of top ranked SNPs, the number of common SNPs among k = 20 random samples is graphed. The observed SNP rank data are shown as orange solid lines and the random SNP rank data are shown as blue dashed lines. The inset shows an enlargement of the original plot for all 24,355 tested SNP, showing that eventually all SNPs are included in the list using either method.

**Fig 3:**
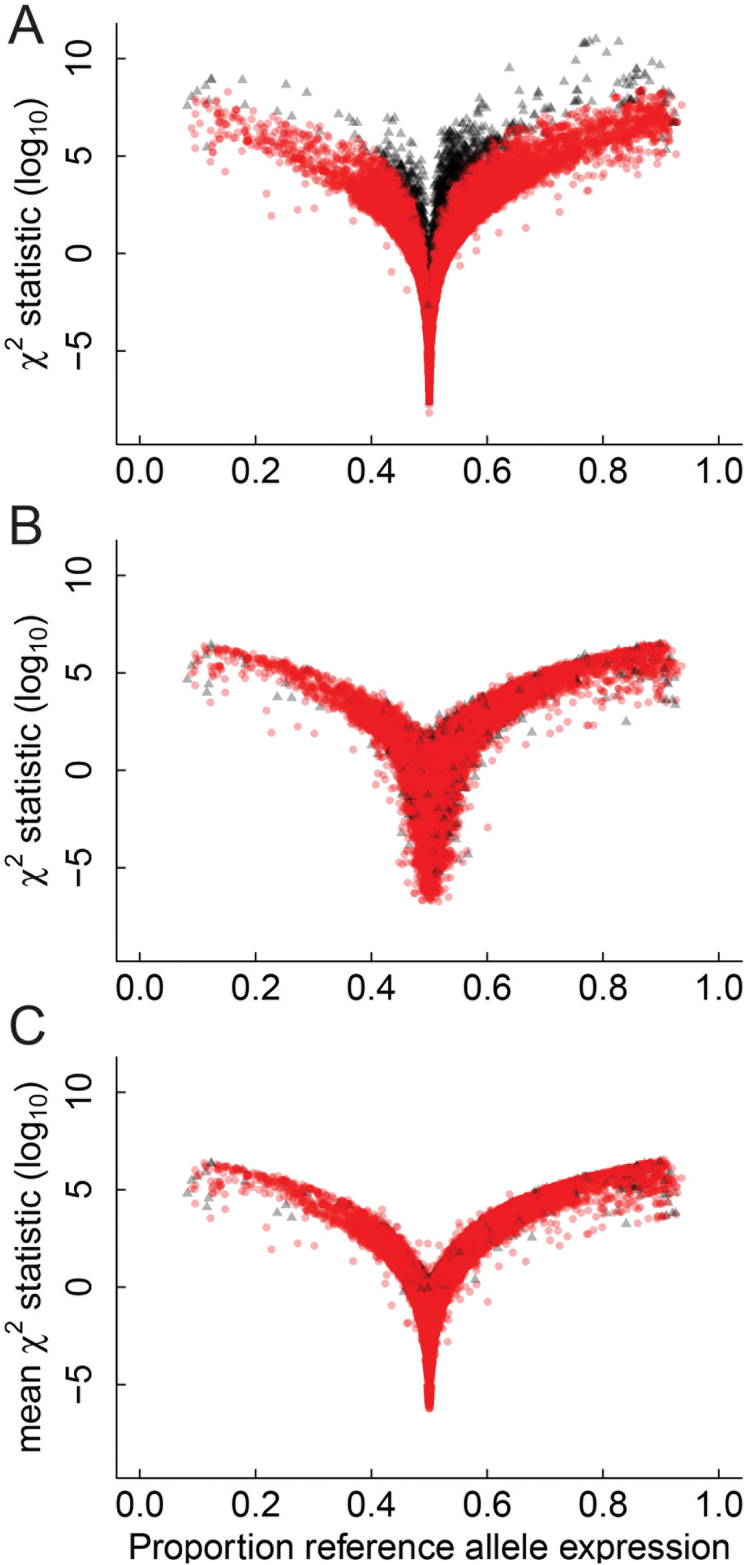
Down-sampling leads to more informative results. The three panels show the relationship between the chi-squared statistic for AI as a function of the proportion of expression of the reference allele. Black triangles represent SNPs associated with highly expressed genes and red circles represent SNPs that are not associated with highly expressed genes. A. No down-sampling. The plot shows that when using the original analysis framework many highly significant SNPs for AI were also highly expressed, indicating an intrinsic bias towards SNPs with higher expression. Highly expressed SNPs were defined as those with overall expression in the top 5% of the entire set of SNPs and are represented by red triangles. B. One round of down-sampling. C. The panel shows the mean test statistics over twenty repetitions of down-sampling and displays stability associated with the down-sampling method by comparison with panel B.

The majority of genes ranking high for AI were also highly expressed (Fig 2A). This result is attributed to the increased statistical power for detecting AI in genes with higher expression due to their increased read counts for each allele and thereby an increased ability to discern smaller levels of AI. Thus, there is an ascertainment bias in the discovery process. We addressed this inherent bias via a down-sampling approach. Down-sampling was carried out at each SNP by randomly sampling reads without replacement to a read depth D, and repeating this k times. The coverage level, D, was chosen by comparing the estimated overall bias and estimated overall variance for differing read depths (Fig 2B). The read depths chosen corresponded to the 5%, 10%, 15%,…, 100% quantiles of the overall expression, displayed as numbers on the curve in Fig 2B. Using this approach, the most suitable down-sampling read depth was with D = 50 reads as it mitigated between the overall bias and overall variance (Fig 2B). The stability of the results of the down-sampling method was also determined as the method is a non-deterministic method relying on random selection. Fig 2C shows the stability of the down-sampling analysis with D = 50 and k = 20, compared to random selection. There were 154 SNPs in the top ranked 200 SNPs for every down-sampling iteration, indicating that the down-sampling method was highly stable compared to random selection of SNPs. Following the implementation of the down-sampling procedure (with D = 50 and k = 20) there was no correlation between highly expressed SNPs, (i.e. with high read coverage) and AI SNP ranking (Figs 2A and 3).

**Fig 4:**
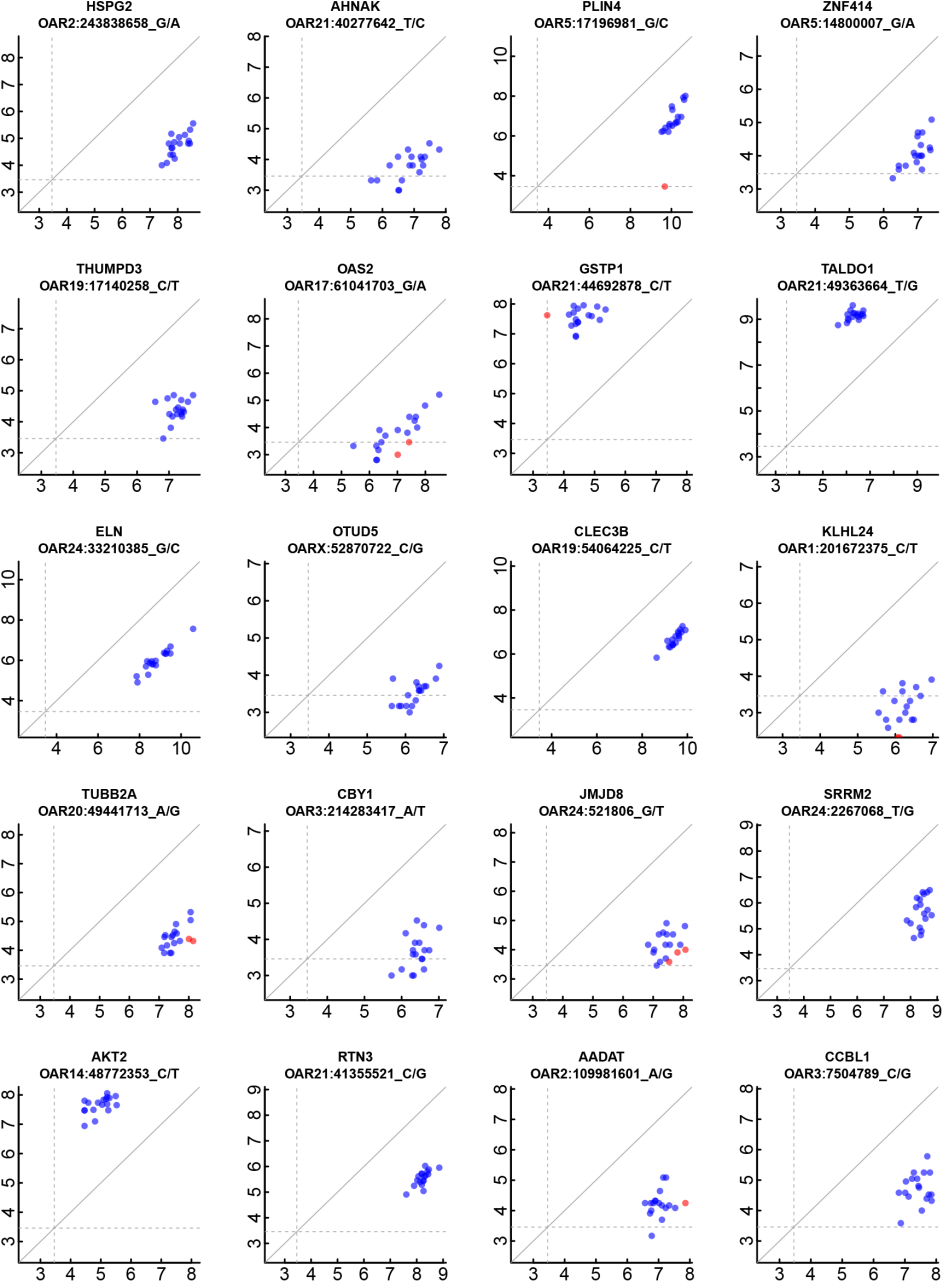
Scatterplots of highest ranked genes. For the 20 top ranked genes for AI, scatterplots of read depths for the reference (abscissa) and alternate (ordinate) alleles are graphed for the highest ranking SNP for that gene. Read depths are transformed into log_2_ (1 + read counts). Also shown for each gene is the gene symbol, genome coordinate and the reference and alternative alleles. Data for all animals are shown, including some that are homozygous at the locus. Blue circles denote individuals with heterozygous marker genotypes. Red circles represent those individuals classified as homozygous and thus were not included in the AI testing. Dashed lines represent minimum expression thresholds. The diagonal line represents allelic balance in gene expression.

The top-ranked 20 genes showing AI after down-sampling are listed in Table 2 and representative scatterplots of read depths for the reference and alternative alleles of these top-ranked genes from informative animals are shown in Fig 4. The complete list of ranked genes showing evidence for AI using the down-sampling method is presented in S4 Table. The table also highlights a subset of 1,293 genes in the top ranked 1,500 genes after down-sampling that were also significant in the primary analysis with P<1.7E-5 (i.e. gene based Bonferroni corrected P<0.05).

**Table 2:** Top ranked 20 genes showing AI after incorporation of the down-sampling approach.

Ranking	Gene	SNP coordinate	Ref	Alt
1	*HSPG2*	chr2:243838658	G	A
2	*AHNAK*	chr21:40277642	T	C
3	*PLIN4*	chr5:17196981	G	C
4	*ZNF414*	chr5:14800007	G	A
5	*THUMPD3*	chr19:17140258	C	T
6	*OAS2*	chr17:61041703	G	A
7	*GSTP1*	chr21:44692878	C	T
8	*TALDO1*	chr21:49363664	T	G
9	*ELN*	chr24:33210385	G	C
10	*OTUD5*	chrX:52870722	C	G
11	*CLEC3B*	chr19:54064225	C	T
12	*KLHL24*	chr1:201672375	C	T
13	*TUBB2A*	chr20:49441713	A	G
14	*CBY1*	chr3:214283417	A	T
15	*JMJD8*	chr24:521806	G	T
16	*SRRM2*	chr24:2267068	T	G
17	*AKT2*	chr14:48772353	C	T
18	*RTN3*	chr21:41355521	C	G
19	*AADAT*	chr2:109981601	A	G
20	*CCBL1*	chr3:7504789	C	G

### Enrichment for imprinted genes

Of the 16 experimentally documented ovine imprinted genes in the GeneImprint database [6], eight were expressed in PRAT and four of these were in the list of 1,293 genes showing AI (i.e. genes in the top ranked 1,500 genes after down-sampling and with Bonferroni P<0.05). These four imprinted genes corresponded to the archetypal multispecies imprinted genes *IGF2* (minimum SNP uncorrected P-value = 1.6E-35; Allelic Imbalance ratio (AI) = 0.34, i.e. ratio of SNP reads to reference genome reads) and *IGF2R* (minimum SNP P-value = 5.1E-121; AI = 0.12) as well as *GNAS* (minimum SNP P-value = 3.7E-171; AI = 0.56) and *GRB10* (minimum SNP P-value = 1.2E-80; AI = 0.78). Interestingly, three of four of these genes are reported to be maternally expressed, the exception being *IGF2*, which is paternally expressed. The ovine genes showing AI (top ranked 1,500 genes after down-sampling and additionally filtered for genes with Bonferroni P<0.05) were also intersected with all known mammalian imprinted genes whose orthologs were expressed in ovine PRAT, revealing 20 genes (bolded in S4 Table). The identification of known ovine and mammalian imprinted genes in the list of genes showing AI in ovine PRAT was consistent with the process used to identify genes more broadly showing AI.

### Enrichment for genes showing AI with murine orthologous genes associated with monoallelic expression

The dbMAE database was used to intersect ovine genes showing AI with murine genes with predicted monoallelic expression as determined by genome-wide chromatin modification analyses [33]. The murine myocyte C2C12 and C2C12_EqS cell lines were used for the analysis as the database contained no adipocyte samples and brown adipocytes and skeletal muscle cells share a common cell precursor [35, 36]. The input for the analysis was the list of 1,293 ovine genes resulting from the top ranked 1,500 genes for AI after the downsizing analysis that were additionally filtered to only include genes with a Bonferroni-corrected P<0.05 for the most significant SNP per gene in the original analysis. The intersection of the two orthologous gene lists revealed a significant 315 gene overlap (P = 7.2E-20; hypergeometric test).

### Functional enrichments for genes showing allelic imbalance in gene expression

**Fig 5:**
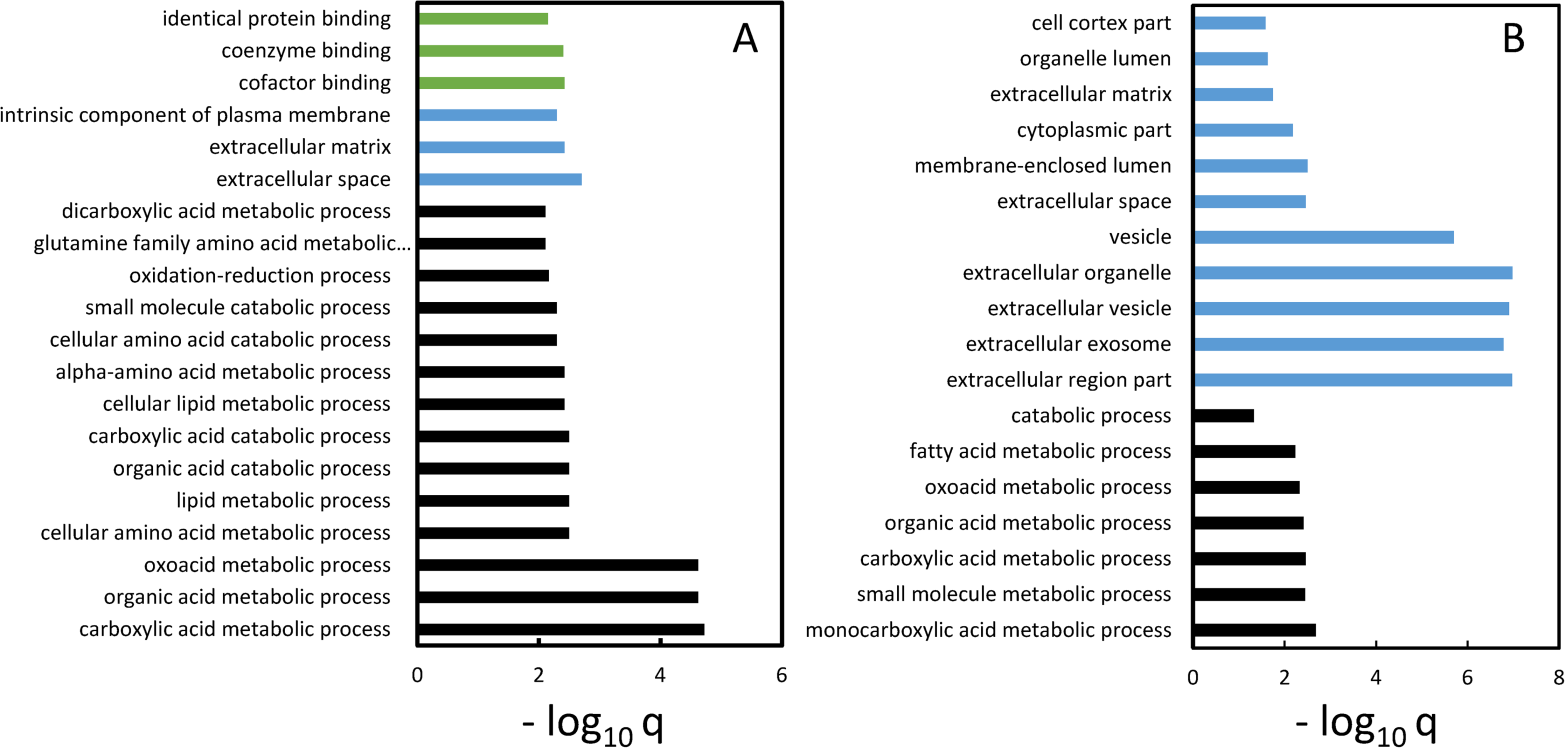
Enriched gene ontology terms for genes showing AI. A. Functional enrichments for gene ontology (GO) terms associated with the down-sampled ranked list of genes showing AI. Only the top ranked 20 terms are shown. Functional enrichments were performed using a Wicoxon rank sum test implemented using GOseq [28]. q values less than 0.05 were considered significant. The GO term categories included Biological Process (black), Cell Component (blue) and Molecular Function (green). B. The top ranked 1,500 genes with AI after the down-sampling analysis were filtered to only include genes that showed significant AI (q<0.05) in the primary analysis. These 1,293 genes were examined for GO term functional enrichments using GOrilla [29] with a background gene list of all of the down-sampled ranked genes(q< 0.05). There were no term enrichments for the Molecular Function category. In both panels the q value for enrichment was -log_10_ transformed. Both analyses used a minimum read count threshold of 10.

Two general approaches were used to examine the functional enrichments associated with the genes showing AI. In the first approach, functional enrichment analysis was performed with the ranked list of genes (after down-sampling) using a Wilcoxon rank sum test implemented with the GOseq R package (FDR<0.05) [28] (Fig 5A). There were three functional themes, lipid metabolism, amino acid metabolism and the extracellular matrix, with each theme represented by multiple enriched GO terms. The first and second themes primarily reflected mitochondrial function. The second approach used the top ranked 1,500 genes showing AI after the down-sampling analysis which were filtered to only include genes that were significant for AI in the original analysis (P<1.7E-5 i.e. Bonferroni corrected P<0.05). This resulted in a list of 1,293 genes that was tested for enriched functional GO terms using GOrilla [29] with a background gene list of all genes tested for AI (q<0.05) (Fig 5B). In terms of biological themes, this analysis generated similar results to the first method i.e. multiple enriched terms for fatty acid/ lipid metabolism (mitochondrial function) and the extracellular matrix. Functional enrichments were also performed with genes showing AI that were independently identified in a similar manner except using a more stringent minimum read count of 30 instead of 10 (i.e. 3,094 down-sampled ranked genes of which 992 were significant at Bonferroni P<0.05) (S5 Table). The enriched GO terms (q<0.05) were similar to the those identified with genes discovered using the minimum read count threshold of 10 (Fig 5). This result reiterated the enrichment for the major biological themes of lipid metabolism, amino acid metabolism and the extracellular matrix. It also demonstrated that the functional enrichment analyses identified similar GO terms at a more stringent threshold for minimum read count for AI gene discovery, thereby further justifying the use of a minimum read count threshold of 10.

The promoters of the 1,293 genes showing AI (minimum coverage threshold of 10) were then examined for enriched (q<0.05) transcription factor binding sites using DAVID [30] (Table 3). Three of the top ranked ten transcription factors (YY1, PPARA and XBP1) have been specifically implicated in the regulation of the transcription of genes involved in core aspects of brown adipose tissue function [41–43]. Moreover, using the Hallmark experimental gene expression datasets in GSEA [31, 32] with the 1,293 AI genes as input, there was enrichment for adipogenesis, fatty acid metabolism and oxidative phosphorylation themes (q = 1.1E-25, 4.6E-15, 5.1E-11, respectively), as well as myogenesis (q = 9.9E-10) in the top ranked ten datasets. Thus, many of the identified genes showing AI in ovine brown adipose tissue are implicated in mitochondrial function, more broadly energetics, and are also expressed in skeletal muscle.

**Table 3:** Transcription factor binding site enrichments^1^ for the top ranked genes showing AI.

Category	Transcription factor	FDR
UCSC_TFBS	YY1	5.57E-18
UCSC_TFBS	PPARA	1.09E-12
UCSC_TFBS	PAX5	1.91E-12
UCSC_TFBS	SRF	1.23E-11
UCSC_TFBS	PAX4	8.41E-11
UCSC_TFBS	HMX1	9.34E-11
UCSC_TFBS	XBP1	1.15E-10
UCSC_TFBS	AP4	3.52E-10
UCSC_TFBS	ATF6	5.05E-10
UCSC_TFBS	NFY	5.45E-10

1 The top ranked 1500 genes showing AI after down-sampling were filtered for genes showing AI in the original analysis at P<1.7E-5 (Bonferroni P<0.05). The promoters of these genes were then examined for transcription factor binding site enrichments using DAVID. Only the top tanked ten transcription factors are listed.

### Identification of ovine QTL associated with genes showing AI

The ovine genes showing AI were intersected with 402 QTL for a variety of lamb production traits relating to fat deposition, muscling and growth derived from the Animal QTLdb database [37]. The input for the analysis was the 1,293 ovine genes resulting from the top ranked 1,500 genes after the downsizing analysis that were additionally filtered to only include genes with a Bonferroni P<0.05 for the most significant SNP per gene in the original analysis. A total of 795 genes (61%) showing AI were present within QTL for 61 traits. Table 4 summarises the traits with the greatest number of QTL (≥6) containing genes showing AI. QTL for two growth related traits, body weight at various ages and average daily weight gain were highly represented. The table also contains a number of muscling related traits and four traits linked to internal fat deposition.

**Table 4:** Traits with the greatest number of QTL containing genes showing AI.

Trait	Number of QTL
Body weight at various ages	17
Average daily weight gain	16
Muscle weight in carcass	12
Body weight at slaughter	11
Hot carcass weight	11
Longissimus muscle area	11
Lean meat yield percentage	10
Fat weight in carcass	8
Carcass fat percentage	7
Muscle density	7
Internal fat amount	6
Total fat area	6

## Discussion

The current investigation used RNA-Seq data from perirenal adipose tissue taken from 18 late gestation fetal lambs to identify genes showing allelic imbalance in gene expression by making use of informative SNP markers at heterozygous loci. Initially, a total of 7,631,907 potential SNPs was identified. This number is consistent with SNP discovery rates in outbred sheep populations [21]. A filtered list of 24,355 SNPs at heterozygous loci within ENSEMBL genes was tested for evidence of AI. The process addressed a number of inherent analytical issues and statistical biases to identify AI rankings for 5,810 genes, from which a conservative subset of 1,293 genes (25.6%) was identified (genes in the top ranked 1,500 genes after downsizing and filtered for Bonferroni P<0.05).

The stringent initial SNP filtering process reduced the number of SNPs from 7,631,907 to 24,355 informative (filtered) SNPs located only at heterozygous loci. The confirmation that 88.4% of the filtered SNPs were also independently present in the dbSNP database indicated that the SNP identification and filtering process was highly robust. A Poisson statistical model was then used to produce a likelihood ratio test for AI associated with each of the filtered SNP. It was demonstrated that SNPs associated with abundant transcripts were more likely to reveal AI due to their increased read depths and hence greater statistical power to detect small differences in allele specific expression. The analysis then controlled for this data acquisition bias by down sampling and ranking of the genes for AI.

The process used for identification of genes showing AI has limitations as its efficiency is dependent on the population genetic structure, its genetic diversity and a minimum level of gene expression. Hence, some genes with AI may not have been assessed. However, based on the tested genes it is concluded that cryptic *cis*-acting genetic variation and to a much lesser extent genomic imprinting and clonally stable monoallelic epigenetic gene silencing have broad impact on allelic imbalance 1,293 genes. Thus, approximately 25% of the tested expressed genes in PRAT showed AI and these may therefore have potential to impact phenotype. This percentage of genes showing AI is consistent with data for a range of bovine tissues from a single individual [44] and indicates that AI is widespread throughout the genome.

*Cis*-acting genetic variation is likely to be the principal cause of most AI in the genome although for a small number of genes AI may result from nongenetic mechanisms such as genomic imprinting [2–6]. Examination of the genes showing evidence for AI in the current analysis identified four genes known to be imprinted in ovine tissues and also 20 genes that are imprinted in other mammalian species. Hence, the identification of these genes provides independent support for the more general process used for the discovery of genes showing AI by all mechanisms. While comprehensive studies using humans and mice have identified 246 and 149 imprinted genes, respectively, far fewer have been identified for sheep and cattle (16 and 31, respectively) [2–6]. The ruminant imprinted gene list may be an underestimate due to the lack of comprehensive investigations. In addition, some imprinted genes are difficult to identify as they show tissue and developmental imprinting specificities and others are characterised by polymorphic imprinting within a population [45]. Thus, the genes showing AI in the current analysis may also include unidentified imprinted genes. The formal proof of the imprinting status of these genes requires additional investigation.

There was also significant overlap of ovine genes showing AI with murine orthologous genes predicted to be monoallelically expressed in two myoblast cell lines through epigenetic mechanisms. The predictions were based on the co-occurrence of two chromatin modifications, H3K27me3 and H3K36me3, in the gene body [33, 34]. The gene overlap between these two different analyses in different species suggests that there is some conservation of genes showing AI, which has also been demonstrated by comparisons between human and murine genes [34]. The gene overlap in the current investigation may reflect conservation of epigenetically driven AI in the absence of causal genetic variations. Alternatively, the overlap between these datasets may indicate the identification of orthologous genes that are particularly susceptible to genetic variation causing gene expression AI. The latter possibility could arise if there was a high number of regulatory elements affecting the expression of each gene and hence a greater chance of somatic and meiotically stable genetic variations in these regions altering gene expression. It is also possible that genetic variation within and near gene regulatory regions for these genes directly alters the extent of chromatin modifications and thereby gene expression.

The enriched mitochondrial and lipid catabolism functions associated with genes showing AI suggest that genetic variants affecting energy use are impacting perirenal adipose tissue function and possibly more broadly other tissues with strong energy demands e.g. skeletal muscle and white adipose tissue. Indeed, many of the genes showing AI in brown adipose tissue were also expressed in skeletal muscle and both tissues are known to be derived from the same progenitor cells [35, 36]. Perirenal adipose tissue, a brown adipose tissue depot at birth, is particularly energy intensive in the neonatal lamb as it protects the new born lamb from hypothermia by decoupling mitochondrial oxidative phosphorylation from electron transfer resulting in the dissipation of the mitochondrial proton-motive force and release of heat (nonshivering thermogenesis) [35, 36, 46]. Although only representing a minor percentage of total body weight, activated brown adipose tissue has the capacity to produce 300 fold more heat/weight of tissue than any other tissue [47]. Accentuating the risk of hypothermia in neonatal lambs is their limited energy reserves and high surface area to volume ratio. Moreover, the rapid growth rate of lambs is also subject to high energy demands.

Genes showing AI may show tissue specific patterning and hence the importance of the genes discovered in brown adipose tissue to production traits involving other tissues is unclear [44]. However, energy intensive production traits such as lamb survival and growth rate may have strong direct contributions from brown adipose and skeletal muscle tissues. These traits are likely subject to strong artificial selection in domestic livestock populations due to their commercial relevance. Hence, genetic variants that impact on these traits may be under selection in genetically diverse sheep populations. The enriched extracellular matrix terms for the genes showing AI indicate that genetic variants in genes contributing to the matrix are present in the population and these may potentially alter cell-cell and cell-matrix interactions. Therefore the capacity for tissue hypertrophy and remodelling during growth may be affected. This potential impact is particularly relevant for hypertrophic cellular responses in adipose tissue and skeletal muscle occurring during rapid postnatal lamb growth.

Many genes showing AI in late gestational fetal brown adipose tissue were positioned within multiple genomic regions associated with QTL for traits associated with lamb growth, muscling and internal fat deposition. Lambs retain small quantities of brown adipose tissue in various anatomical locations that could directly contribute to the internal fat deposition traits and indirectly other traits through its strong metabolic activities [46, 48]. Moreover, brown adipose tissue cells and skeletal muscle cells share a common progenitor cell origin [35, 36] and both have related gene expression programs as demonstrated by functional enrichment analysis (Hallmark experimental datasets in GSEA). Thus, there may be direct impact of the discovered AI genes in skeletal muscle as well as brown adipose tissue. There are some limitations associated with the QTL and AI gene intersection analysis as the collective genomic coverage of the investigated QTL was substantial (19.3% of the genome). Hence, finer resolution of the identified QTL is required to narrow the number of candidate genes showing AI that may underlie the identified QTL.

Genetic variation in gene regulatory elements is a major contributor to variation in complex traits [49, 50]. More specifically, genetic variation in regulatory elements located distally of genes and within gene promoters can modulate gene expression [51]. Thus, genetic variation in gene promoter regulatory regions that alters gene expression may contribute to variation in complex traits within outbred populations. The genes showing AI were associated with a number of QTL for a variety of production traits likely impacted by brown adipose tissue and skeletal muscle deposition and function. There was also enrichment for specific transcription factor binding sites in the promoters of genes showing AI, particularly transcription factors known to impact brown adipose tissue function and energy homeostasis. This result hints that regulatory regions associated with these transcription factor binding sites may be priorities for future investigations to potentially identify causal genetic variants contributing to gene expression AI in the brown adipose tissue samples from the investigated sheep population and more widely their potential contribution to production trait QTL.

## Conclusions

Genetic variation in a population of sheep is responsible for considerable allele specific gene expression imbalance, which may be associated with energy intensive production traits, such as neonatal survival and growth, and traits involving strong postnatal tissue remodelling and cellular hypertrophy e.g. adipose and skeletal muscle deposition. The marker SNPs associated with AI may therefore have value for DNA marker-assisted selective breeding in the sheep industry. The identification of the causal *cis*-acting SNPs, whilst in the vicinity of genes showing AI, requires further investigation. It is possible that some of the causal SNPs are represented in the SNP markers used to measure AI.

## Supporting information

S1 Fig Boxplots for RNA-Seq data for all samples. Boxplots of uniquely mapped paired end sequence read counts covering the filtered SNPs for the 18 biological samples are presented. The ordinate is the log_10_ transformed read counts. The horizontal line shows the median for a sample while the box boundaries indicate the first and third quartiles of the distribution. The 18 samples show similar distributions of read counts.
(TIF)

S2 Fig Effects of the minimum read coverage threshold on the discovery of SNPs in genes. The minimum read cover threshold was varied between 5 and 100, and its impact on the number of filtered SNPs (top panel), number of genes tested for AI (middle panel), and the percentage of filtered SNPs present in dbSNP (bottom panel) are plotted. A minimum coverage threshold of 10 (larger blue circle) was selected for most analyses.
(TIFF)

S1 Table Read mapping statistics for each sample. (DOCX)

S2 Table Contingency table of SNPs that passed the filtering criteria and were present in dbSNP. (DOCX)

S3 Table Poisson-based likelihood ratio test (LRT) for the 24,355 filtered SNPs. (XLSX)

S4 Table Rankings of all tested genes for AI using down-sampling analysis and identification of a subset of the top ranked 1,500 genes that were significant for AI (Bonferroni P<0.05). (XLSX)

S5 Table Enriched GO terms for genes ranked for AI using a minimum read count threshold of 30. (XLSX)
