# Supplementary figures and images for: Gene expression allelic imbalance in ovine brown adipose tissue impacts energy homeostasis
Source: PLoS One. 2017 Jun 30;12(6):e0180378. doi: 10.1371/journal.pone.0180378 (PMC5493397; doi:10.1371/journal.pone.0180378)

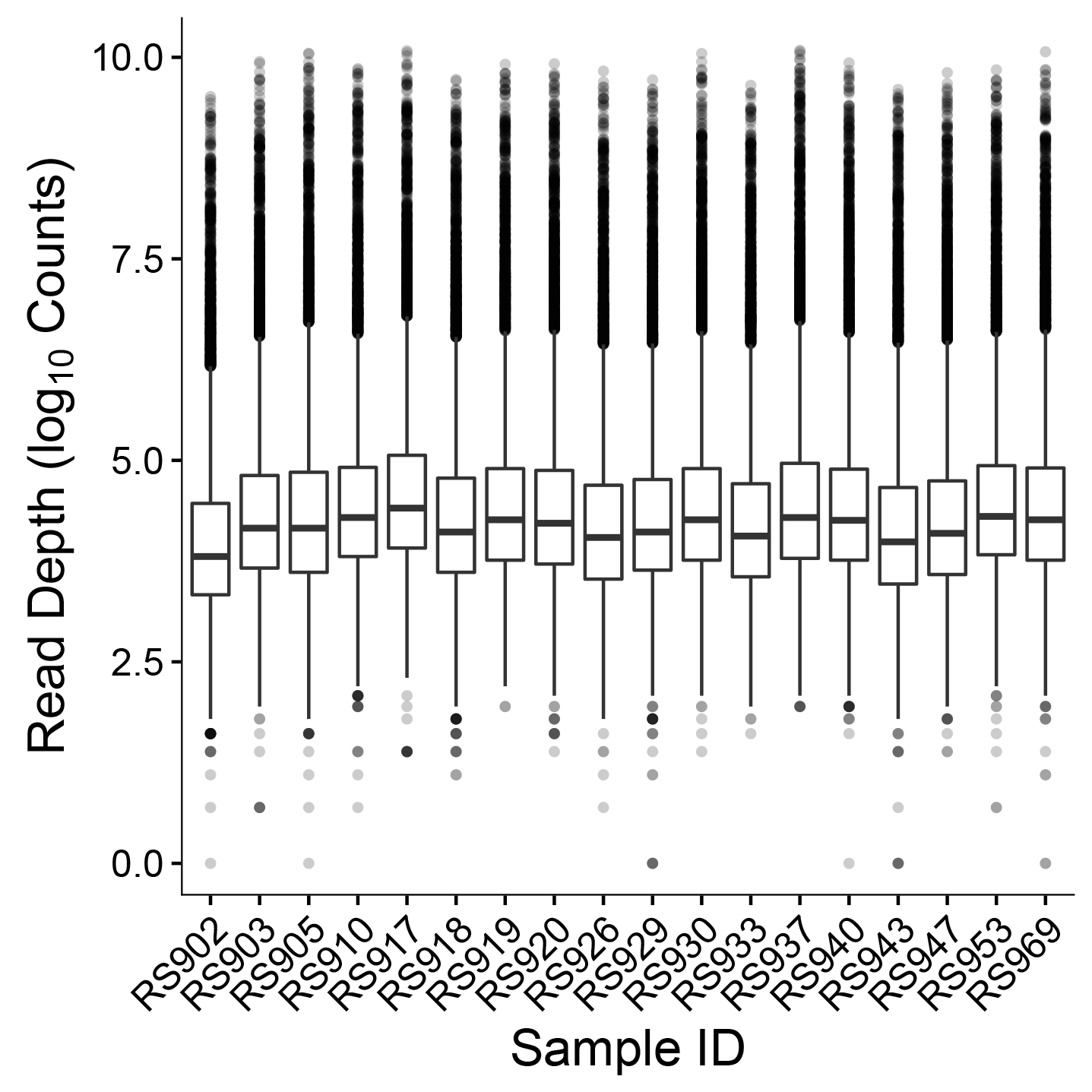

Supplement: S1 Fig — Boxplots of uniquely mapped paired end sequence read counts covering the filtered SNPs for the 18 biological samples are presented. The ordinate is the log10 transformed read counts. The horizontal line shows the median for a sample while the box boundaries indicate the first and third quartiles of the distribution. The 18 samples show similar distributions of read counts. (TIF) [file pone.0180378.s001.tif]

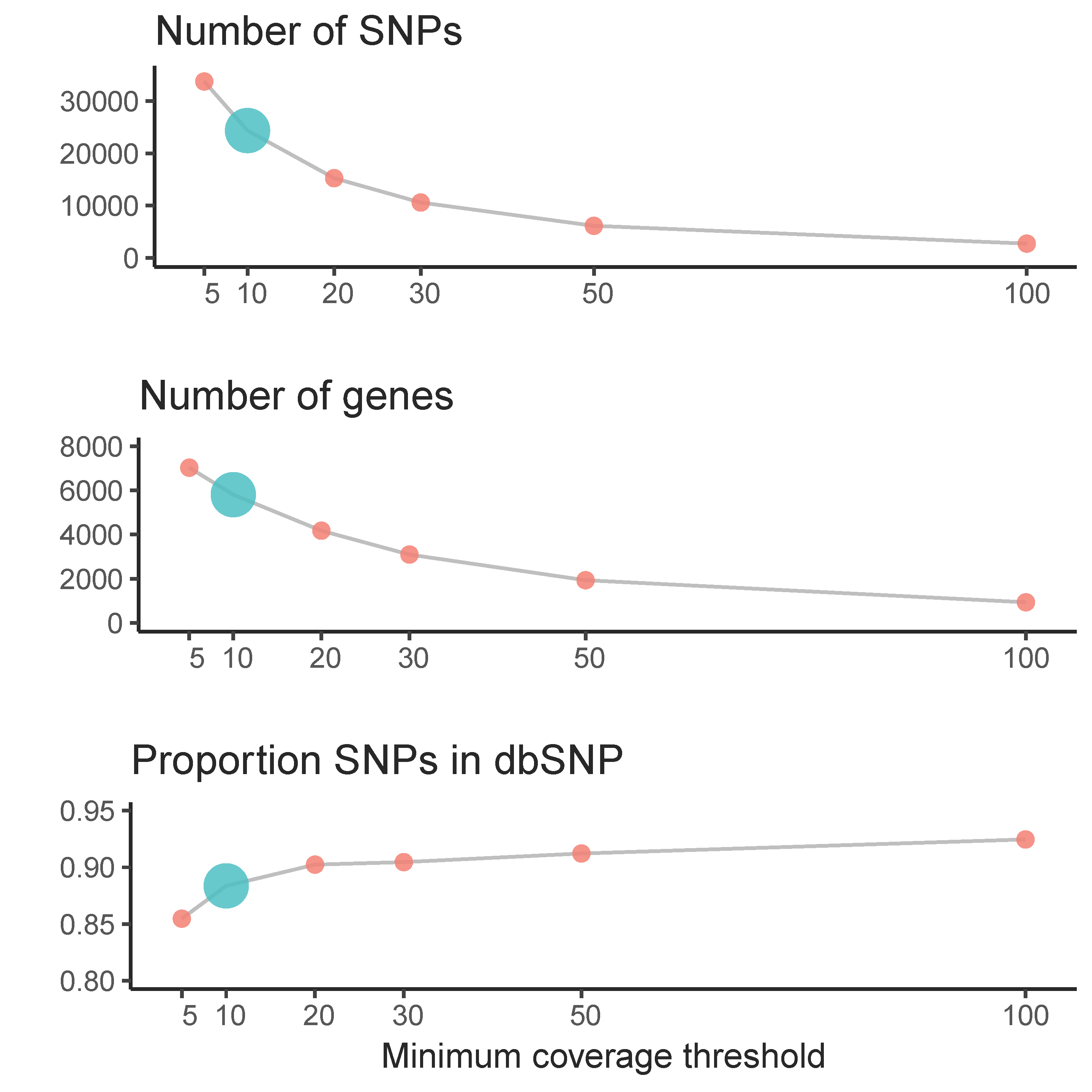

Supplement: S2 Fig — The minimum read cover threshold was varied between 5 and 100, and its impact on the number of filtered SNPs (top panel), number of genes tested for AI (middle panel), and the percentage of filtered SNPs present in dbSNP (bottom panel) are plotted. A minimum coverage threshold of 10 (larger blue circle) was selected for most analyses. (TIFF) [file pone.0180378.s002.tiff]
