# Supplementary material for: Gene expression allelic imbalance in ovine brown adipose tissue impacts energy homeostasis
Source: PLoS One. 2017 Jun 30;12(6):e0180378. doi: 10.1371/journal.pone.0180378 (PMC5493397; doi:10.1371/journal.pone.0180378)
Supplement: S1 Table — (DOCX) [file pone.0180378.s003.docx]

**Table S1**. Read mapping statistics for each sample.

| Sample ID | Input read number | Uniquely mapped read number^1^ | % |
| --- | --- | --- | --- |
| PS910  RS902  RS903  RS905  RS917  RS918  RS919  RS920  RS926  RS929  RS930  RS933  RS937  RS940  RS943  RS947  RS953  RS969 | 40,079,468  26,841,667  36,543,894  39,210,314  46,251,180  36,176,568  39,566,696  40,414,417  33,087,112  36,158,902  40,264,154  32,383,590  45,370,183  41,683,149  31,968,946  33,803,703  39,609,081  41,371,752 | 29,021,746  18,370,208  25,933,048  26,833,435  32,077,318  25,006,646  27,812,920  26,966,797  23,017,579  24,378,104  28,097,684  22,687,453  30,714,067  28,165,815  21,909,286  23,602,327  28,541,931  28,268,296 | 72.4  68.4  70.9  68.4  69.4  69.1  70.3  66.8  69.6  67.4  69.8  70.1  67.7  67.6  68.6  69.8  72.1  68.3 |

1. Reads were deemed successfully mapped if both paired reads were uniquely mapped to the reference genome.
