# Supplementary material for: Gene expression allelic imbalance in ovine brown adipose tissue impacts energy homeostasis
Source: PLoS One. 2017 Jun 30;12(6):e0180378. doi: 10.1371/journal.pone.0180378 (PMC5493397; doi:10.1371/journal.pone.0180378)
Supplement: S2 Table — (DOCX) [file pone.0180378.s004.docx]

**Table S2**: Contingency table of SNPs that passed the filtering criteria and were present in dbSNP

|  | SNP retained | SNP not retained | Sum |
| --- | --- | --- | --- |
| not in dbSNP | 2,831 | 4,690,229 | 4,693,060 |
| in dbSNP | 21,524 | 2,917,323 | 2,938,847 |
| Sum | **24,355** | 7,607,552 | 7,631,907 |
